# Seasonal variation in geographical access to maternal health services in regions of southern Mozambique

**DOI:** 10.1186/s12942-016-0074-4

**Published:** 2017-01-13

**Authors:** Prestige Tatenda Makanga, Nadine Schuurman, Charfudin Sacoor, Helena Edith Boene, Faustino Vilanculo, Marianne Vidler, Laura Magee, Peter von Dadelszen, Esperança Sevene, Khátia Munguambe, Tabassum Firoz

**Affiliations:** 1 Department of Geography, Simon Fraser University, RCB7106 8888 University Drive, Burnaby, BC V5A1S6 Canada; 2 Department of Surveying and Geomatics, Midlands State University, Gweru, Zimbabwe; 3 Centro de Investigação em Saúde de Manhiça, Manhica, Mozambique; 4 Department of Obstetrics and Gynaecology, University of British Columbia, Vancouver, Canada; 5 Department of Obstetrics and Gynaecology, St George’s, University of London, London, UK; 6 Department of Medicine, University of British Columbia, Vancouver, Canada

**Keywords:** Maternal health services, Geographical access to care, Global health, Health geography, Geographical information systems

## Abstract

**Background:**

Geographic proximity to health facilities is a known determinant of access to maternal care. Methods of quantifying geographical access to care have largely ignored the impact of precipitation and flooding. Further, travel has largely been imagined as unimodal where one transport mode is used for entire journeys to seek care. This study proposes a new approach for modeling potential spatio-temporal access by evaluating the impact of precipitation and floods on access to maternal health services using multiple transport modes, in southern Mozambique.

**Methods:**

A facility assessment was used to classify 56 health centres. GPS coordinates of the health facilities were acquired from the Ministry of Health while roads were digitized and classified from high-resolution satellite images. Data on the geographic distribution of populations of women of reproductive age, pregnancies and births within the preceding 12 months, and transport options available to pregnant women were collected from a household census. Daily precipitation and flood data were used to model the impact of severe weather on access for a 17-month timeline. Travel times to the nearest health facilities were calculated using the closest facility tool in ArcGIS software.

**Results:**

Forty-six and 87 percent of pregnant women lived within a 1-h of the nearest primary care centre using walking or public transport modes respectively. The populations within these catchments dropped by 9 and 5% respectively at the peak of the wet season. For journeys that would have commenced with walking to primary facilities, 64% of women lived within 2 h of life-saving care, while for those that began journeys with public transport, the same 2-hour catchment would have contained 95% of the women population. The population of women within two hours of life-saving care dropped by 9% for secondary facilities and 18% for tertiary facilities during the wet season.

**Conclusions:**

Seasonal variation in access to maternal care should not be imagined through a dichotomous and static lens of wet and dry seasons, as access continually fluctuates in both. This new approach for modelling spatio-temporal access allows for the GIS output to be utilized not only for health services planning, but also to aid near real time community-level delivery of maternal health services.

## Background

Geographical proximity to health facilities is a known determinant of both access to maternal care [1–3] and better maternal outcomes [4, 5]. These improved outcomes have been attributed to improved access and utilization of antenatal care, as well as of delivery in health facilities that have skilled birth attendants [6–8].

Quantifying geographic access to care is the most common use of geographical information systems (GIS) in maternal health research and practice [9, 10]. The information obtained can aid in the planning and design of health services by evaluating the geographic reach of the health system to its intended population [11], and showing how underserved regions can be reached [12]. GIS modelling of access to care requires information about the location of health facilities, the geographic distribution of populations [13], and how the population moves to access care.

There is a dearth of research on spatio-temporal modelling that incorporates the component of time to account for changes in access during seasons. This is relevant for all places that are prone to severe weather, and is particularly relevant to sub-Saharan Africa where substantial rainfall and flooding in the wet seasons affect the accessibility of roads at very grand scales [14, 15]. Whether or not these reduced levels of access to facilities may be associated with the seasonal nature of some severe maternal morbidities (such as eclampsia) in tropical climates is unclear [16, 17]. Although women may become sicker in the wet season and attend care later [16, 17], it is clear that these women need emergency obstetric care when it is least likely to be accessible.

Many of the models for potential spatial access to maternal care have been developed in high-income settings and cannot be applied directly to low-income regions. For example, in high-income settings, the 1-h driving time threshold is used as a gold standard for identifying populations that are underserved by the health care system. While a 1-h travel time to care is clinically important in high- or low-income settings, many people in sub-Saharan Africa do not drive cars to access maternal care services [15]. Many women usually in sub-Saharan Africa walk to health centres, or use public transport which represents a mix of walking and driving modes [18, 19]. Furthermore, most of the models for quantifying spatial access to maternal care have not accounted for the impact of seasonality on impeding access to maternal care services. One such study from sub-Saharan Africa [14] presented results in the form of static maps, making it difficult to ascertain how the results could be operationalized and incorporated into health promotion programs that reflect the daily experiences of women as they travel to seek care.

**Fig. 1:**
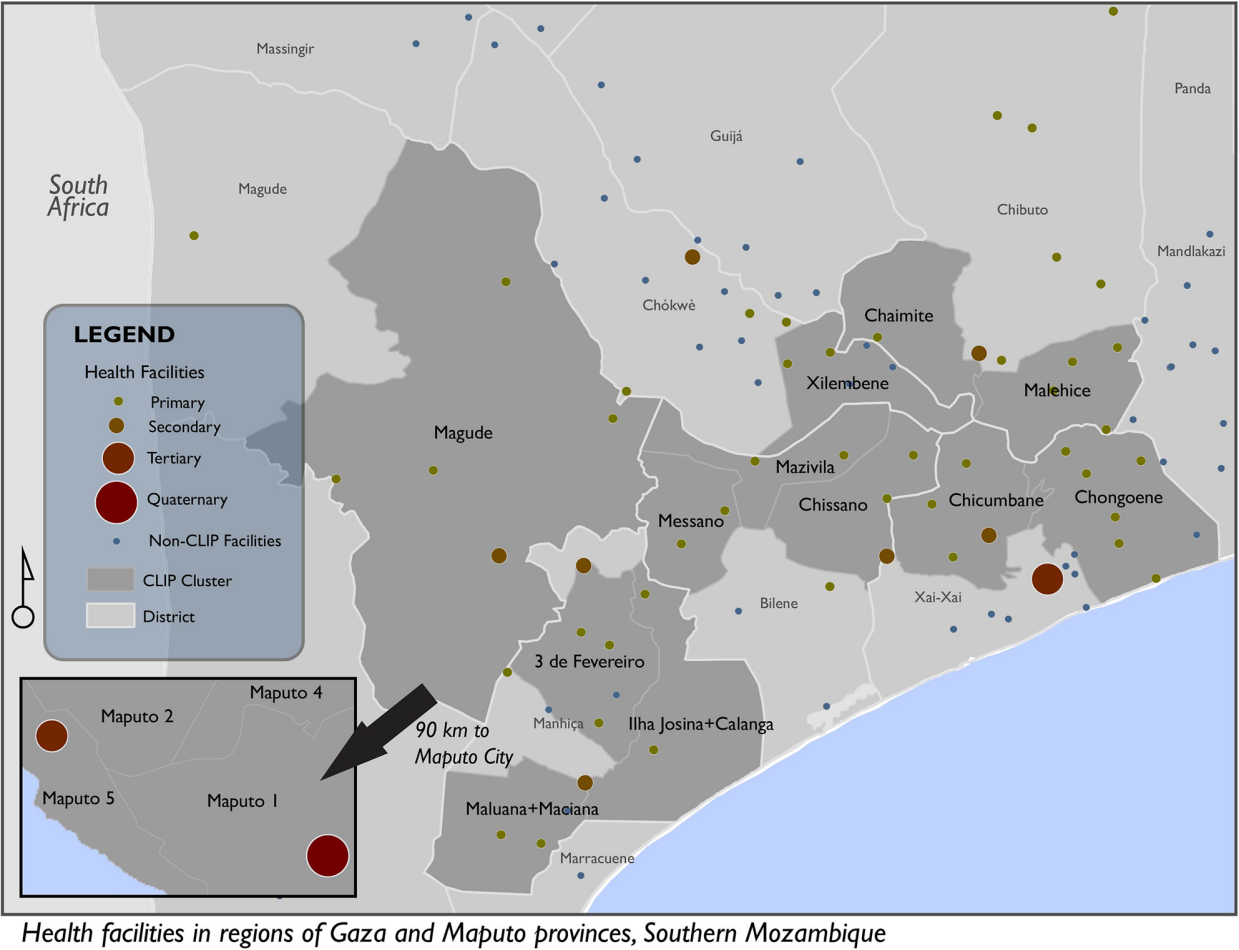
Study site and location of health facilities

This study aimed to extend current models for access to maternal care services by accounting for the impact of flooding and precipitation, making them more relevant for a typical low- or middle-income country context. We developed a spatio-temporal model to describe how women of reproductive age in regions of southern Mozambique (Fig. 1) would potentially access all maternal health care services, by various modes of transport, and how that access may change during different seasons. Mozambique has reported estimates for Maternal Mortality Ratio (MMR) that range from 249 to 480 deaths per 100,000 live births, and is among the top 20 countries with the highest MMRs in the world [20–22]. There has been between 50 and 63% reduction in MMR from since 1990 according to known estimates, land this is sargely due to falling rates of maternal deaths from direct obstetric causes [20]. Nonetheless, much of existing geographical coverage of basic and comprehensive emergency obstetric care (BEmOC and CEmOC) services, which are delivered largely through secondary and tertiary level facilities remains inadequate for the population’s needs [23, 24].

The impact of severe weather on the poor road infrastructure has been described as a barrier for women to seek both antenatal and obstetric care in this region of Mozambique [25]. Flooding in the study area had also previously resulted in entire neighbourhoods being displaced or isolated from health care facilities, with pregnant women in some instances being cut off from emergency obstetric care for months [26]. While according to the authors’ knowledge this is the first study in Mozambique to model geographical access to care based on travel time, previous studies have reported very long distances to access maternal health services and that this has an effect of elevating risk for adverse maternal outcomes [21, 25]. Nonetheless, in other sub-Saharan settings similar to Mozambique, it is known that some women travel for a very long time, sometime up to 2 days to access basic maternal care [14, 27].

## Methods

### Study setting

This study was conducted as part of the feasibility study in preparation for the Community Level Interventions in Pre-eclampsia Trial (CLIP, NCT01911494) in Mozambique [28]. CLIP is an ongoing community-based cluster randomized control trial, aimed at reducing all cause maternal and perinatal mortality and morbidity in the intervention areas. Ethics approval for CLIP was obtained from the CISM Institutional Review Board (CIBS—CISM), the Mozambique National Committee for Bioethics (CNBS) and the UBC Review Board, while approval for the mapping component was also acquired from the Simon Fraser University research ethics board.

### Data

#### Women of reproductive age population and pregnancies

GPS points representing the households where all women of reproductive age in the study area lived were captured from the CLIP baseline census, and were used to determine the spatial population distribution. Characteristics of women of reproductive age within each household were also recorded and include pregnancies within the 12 months prior to the baseline census, completed pregnancies, pregnancy outcomes. These data were used to determine where populations that required access to maternal health services lived, as well as total number of pregnancies and women of reproductive age in different communities.

#### Precipitation and floods

**Fig. 2:**
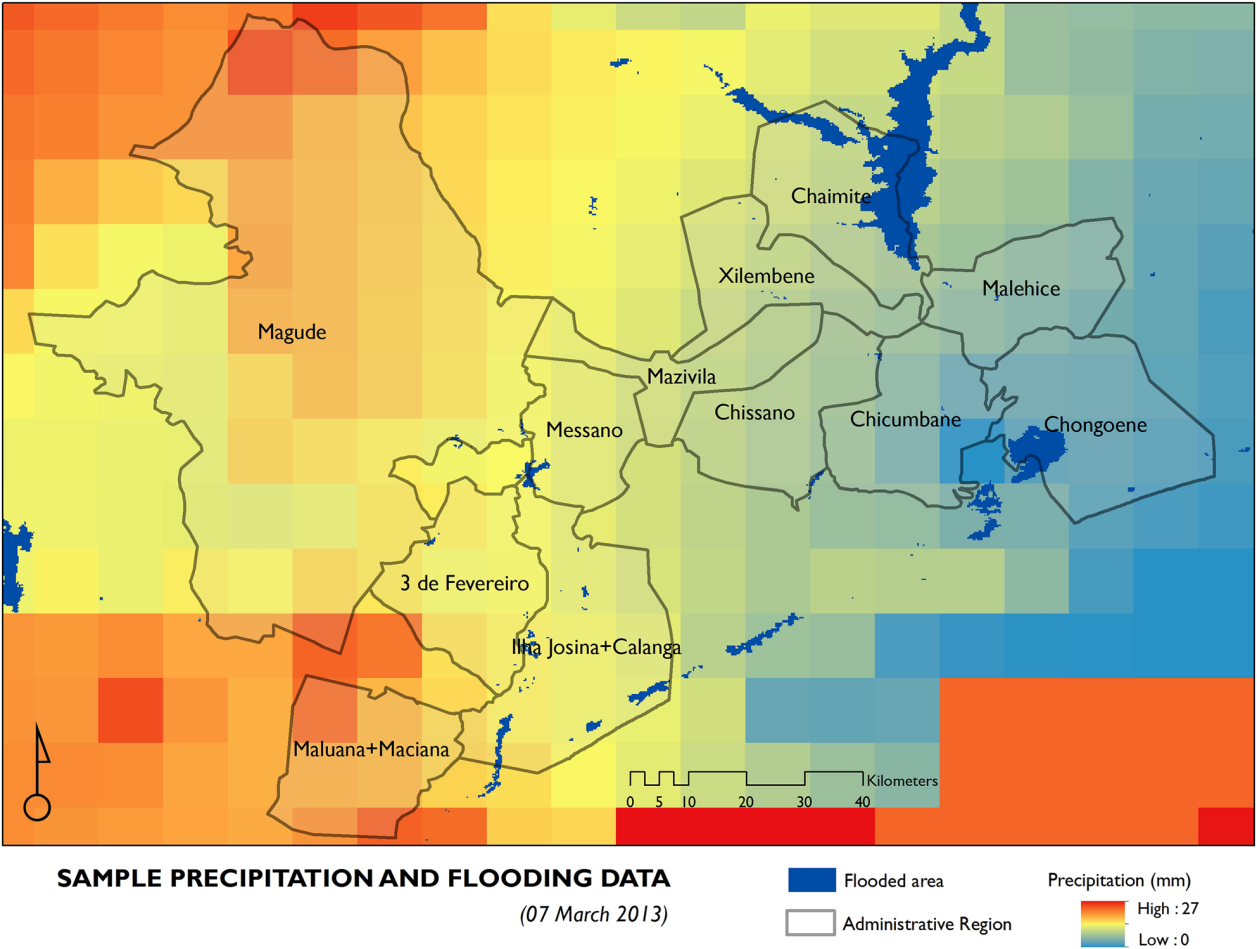
Sample precipitation and flooding data

To account for the impact of adverse weather events we sought to use empirical records of precipitation and floods (Fig. 2). Daily raster records of precipitation within the study area from March 2013 to October 2014 were acquired from the Famine Early Warning Systems Network [29]. These precipitation data are generated on a daily basis using satellite imagery and ground rain gauges [30, 31]. This specific timeline was chosen to coincide with the timeline of the CLIP baseline census. Daily flood extent raster data for the same period was acquired from the Global Flood Observatory [32]. These are also generated on a daily basis from the MODIS satellite [33]. All the required precipitation and flood data were available except for 25 days of flood data and 4 days of precipitation data. These datasets were combined as a first step for creating an impedance surface used to estimate the effect of precipitation and floods on reducing access to health centres. Impedance surfaces are a grid based geographical representation of the ease of traversing through space, with high speed features such as highways, taking less time to traverse when compared to lower speed features such as footpaths for the same unit distance [34]. We assumed that flooded areas were not navigable by any means of transport, while road segments that had precipitation above 1 mm, based on the rainy day threshold [35], would have had reduced maximum travel speeds, as expressed in Table 1.

**Table 1:** Impact of precipitation on speed limits

Precipitation	Road type	Speed limits (km/hr)		
		Driving	Walking	Public transport
Dry weather	Highway	120	5	120
	Paved major road	80	5	80
	Unpaved major road	60	5	60
	Paved minor road	40	4	4
	Unpaved minor road	20	4	4
	Trail	4	3	3
Wet weather	Highway	96	4	96
	Paved major road	64	4	64
	Unpaved major road	42	4	42
	Paved minor road	32	3	3
	Unpaved minor road	14	3	3
	Trail	2.8	2	2
Flood	Impassable (travel time = 99,999,999)			

A new geographical dataset of average weekly rainfall was created from the daily precipitation data. This shift in temporal scale was to account for the impact of precipitation beyond the day it occurred. The 1 mm rainy day threshold (calculated as a weekly average) was used to determine whether a week was to be classified as a rainy week.

#### Road network

An initial road network dataset was provided by the Mozambique National Cartography and Remote Sensing Centre CENACARTA. These data constituted mainly of highways and a few major paved roads and were therefore highly inadequate for the community-level analysis that was intended for this study. We also considered open street map data [36] for the study areas but found it to be inadequate for modeling spatial access at the intended scale due to many missing roads at the community level. Data gaps were filled through a process of manual digitization of the missing roads from a high resolution Bing Maps satellite image service [37]. These roads were classified into highways, major paved roads, major unpaved roads, minor paved roads, minor unpaved roads and trails. A separate verification process was done by staff at CENACARTA and two other independent reviewers to identify and correct instances of misclassification, missing roads and other geometric errors.

Each road segment was assigned a value for travel time based on the length of the road segment and the speed limit. The speed limit was dependant on the road type, whether there was precipitation above the 1 mm weekly threshold, and if the road segment had been classified as being flooded at the time, with precipitation inducing a 20 and 30% reduction in the speed limit on paved roads and unpaved roads respectively. The estimates for the impact of precipitation on speed limits were derived from previous studies [14, 38–41] and are summarised in Table 1.

#### Heath facilities

The GPS coordinates of all public health facilities (Fig. 1) in the country were acquired from the Ministry of Health in Mozambique and research partners at Manhiça Research Centre. These facilities were classified into primary health centres (PHCs), secondary health facilities (SHF) and tertiary health facilities (THF). Data from a 2014 assessment of public health facilities that was conducted as part of the feasibility study for the CLIP trial were used to alter that classification of some of the facilities acquired from the Ministry, because the CLIP facility assessment had more recent results. None of the facilities outside the study area were reclassified due to a lack of recent data. Data on transport options available at each facility was also acquired from the CLIP facility assessment and used as the basis for deciding the most likely mode of transport that women would use to navigate through the facility referral chain.

### Modeling access to care

#### Transport options

Three modes of transports were considered for this project; walking, driving or using public transport. Both walking and driving were treated as single transport modes. However, for public transport we modelled travel assuming that women would walk to the nearest major road to access transport [25], and then drive from that point on. Therefore, for the public transport option we used the same speed limits for travelling through minor roads and trails as we did for walking, but changed the speed limits to be the same as driving for major roads and highways (see Table 1).

The likely scenarios of travel from the home to PHC and subsequent levels of the health care system were determined from the CLIP facility assessment and baseline census. During the facility assessment, information was recorded pertaining to the transport options available at each facility for patient referrals. Data on the personal transport options, as well as transport plans in the event of pregnancy related emergencies were also recorded for every household included in the baseline census and used to decide on the most likely characteristics of the women’s journeys to access maternal care.

#### Modeling spatio-temporal variation in access to care

**Fig. 3:**
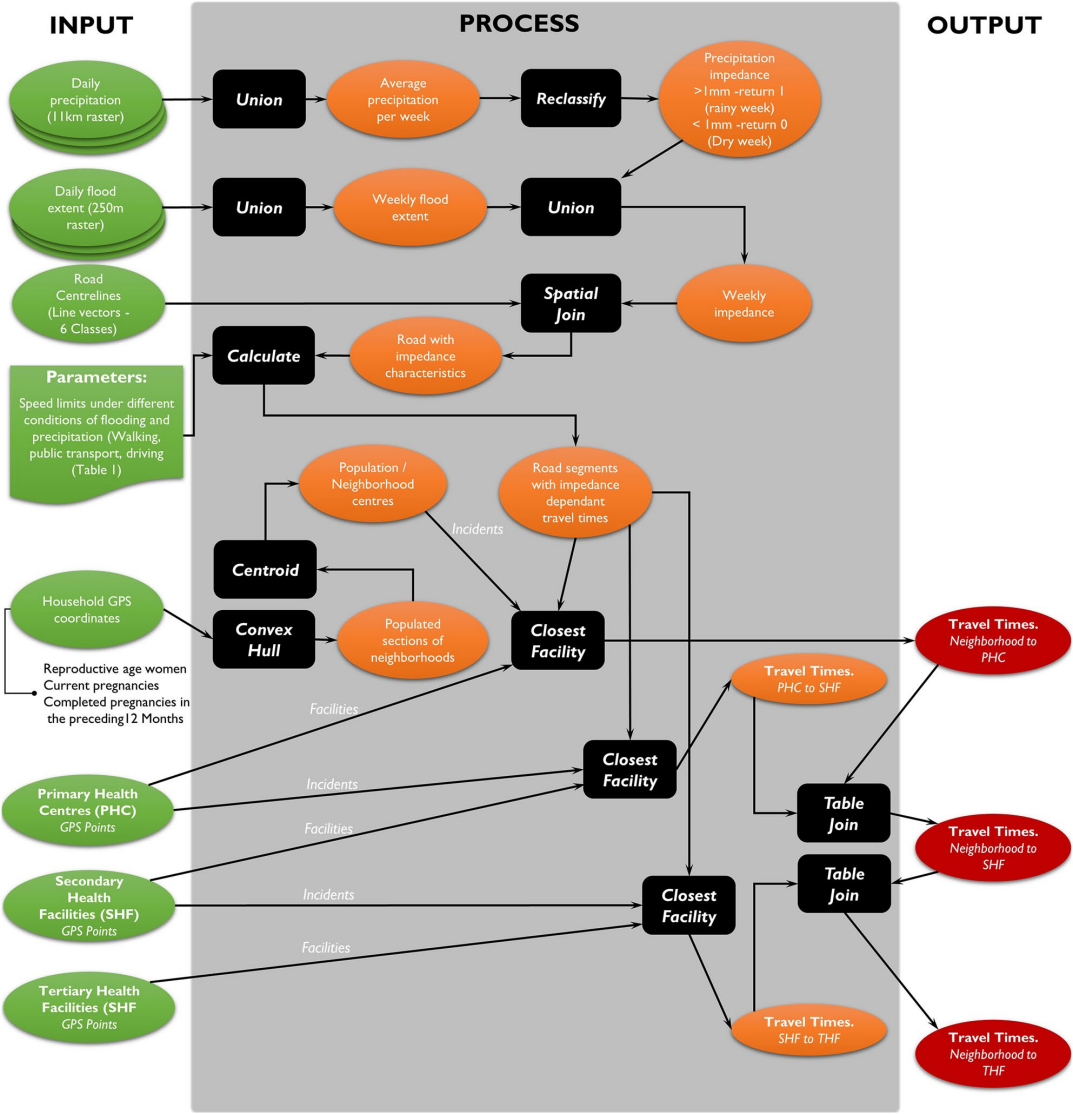
Modeling process for calculating potential spatial access to maternal care services

The process for modeling spatio-temporal variation in access to care is illustrated in Fig. 3. Access to care was modelled from the central location of the populated regions within all the neighbourhoods, instead of the commonly used centre of the actual neighborhood boundaries, which would include uninhabited regions including forests and agricultural zones. The model was developed to estimate travel times from these population centres of 417 neighbourhoods to the nearest health facility accounting for multiple modes of transport, and how this changed overtime. We assumed that most of the population would navigate through the referral chain in a hierarchical sequence; i.e. from home to PHC to SHF then THF. Most higher-level facilities will have lower level ones on premise (e.g. SHFs will normally have PHCs), thereby adopting multiple classifications. This design of the health care system therefore somewhat accounts for instances where people may bypass lower levels of the health care system by going directly to facilities that are closest to them.

The closest facility tool in the ArcGIS software was used to calculate the quickest route between neighborhoods and facilities based on the predefined speed limits along the road network dataset [42] for each of the 87 weeks of the study. Speed limits depended on the impedance values imposed on the road by the road type, precipitation and floods as illustrated in Table 1. The service area tool [43] was used to create cartographically generalized visualizations illustrating the change in spatial access throughout the study at a macro scale.

Once the quickest travel times to the different level facilities for all travel scenarios were calculated for each week in the study timeline, the data was organized into 4 quartiles, for each week, indicating the travel times at the 25th, 50th, 75th and 100th percentiles for all neighborhoods. This exploratory process was done to highlight the disparities that existed in travel times.

We also compared the travel times on the best day in the dry season and the worst day in the wet season to evaluate the extreme impact of precipitation and flooding on access to maternal care for women of reproductive age in general, and for those whom were likely to have been pregnant during these times. Given that it is impossible for women that were registered as having been pregnant during the study to have been pregnant for the entire timeline, we estimated the number of pregnancies at any given time assuming equal likelihood of being pregnant throughout the timeline. The 1- and 2-h travel time thresholds were used for primary care facilities and all other higher level facilities (SHF and THF) respectively, to differentiate women’s access to basic maternal and antenatal care from life-saving care delivered through BEmOC and CEmOC facilities at SHF and THF respectively.

#### Determining isolation of communities

**Fig. 4:**
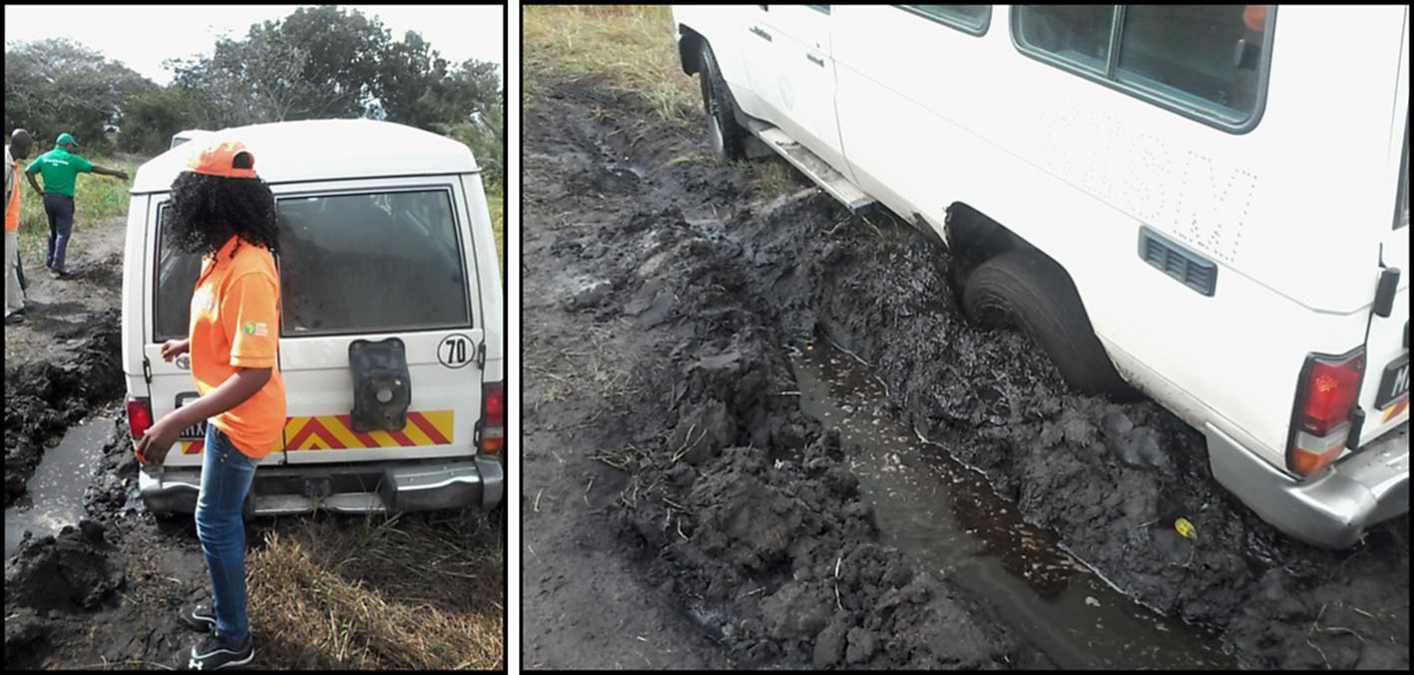
Illustration of the effect of precipitation on hampering transport on minor unpaved roads—Photo taken by field team during a field visit to Calanga, in Maputo province

Communities that would have been totally isolated from health care services because of flooding were also identified. Isolation was when the total travel time to the nearest facility was greater than 99,999,999 min, which was the total time assigned for travelling through a flooded road segment that would have essentially been impassable using motorized transport or on foot. Approximately 85% of the roads in the study area are unpaved, with 75% of these being classified as minor unplanned roads [37]. The road infrastructure is thus typically hard to traverse in the wet season and not usable when flooded (Fig. 4). As the network algorithm used in this study identified the optimum routes with the quickest possible time for traversing from a community to a health facility, instances where travel times were greater than 99,999,999 indicated that no alternate route existed, meaning the road infrastructure could not be used.

### Transport options

According to the baseline census, most women of reproductive age in the study area were likely to either walk or use public transport to travel to the nearest primary health centre (Table 2). This is since 70% of all households indicated not having private transport, and almost 72% of household indicated that pregnant women would nonetheless have access to transport funds when needed.

**Table 2:** Transport options available based on the CLIP baseline census and facility assessment

Facility level	Transport options	*N*	%	Most likely mode of transport
Community to PHC	Number of households that do not own private cars	35,368	69.8	Most women will either 1. Walk to PHC (based on 70% not having personal transport) 2. Use public transport to PHC (based on 72% having access to transport funds)
	Number of households with a bicycle	10,521	20.8	
	Number of households with a motorbike	972	1.9	
	Number of households with a boat	87	0.2	
	Number of households with a car	2847	5.6	
	Number of households with other forms of transport	784	1.5	
	Number of households where pregnant women have access to money for transport	36,648	72.4	
	Number of households that reported that they would get transport help from neighbors or family	6787	13.4	
PHC to SHF	PHCs with functional ambulance or other vehicle for emergency	2	4.0	Most women are likely to be driven from primary to secondary facilities
	PHCs with transport for patients referred to another facility	43	93.0%	
	PHCs with access to an ambulance or other vehicle from another facility	44	96.0%	
SHF to THF	SHFs with functional ambulance or other vehicle for emergency	7	100.0%	Most women are likely to be driven from secondary to tertiary facilities

According to the facility assessment, most women were likely be driven from primary care facilities to higher level facilities. Women were either driven by ambulance from secondary to tertiary facilities, or by private cars from primary health centres to secondary facilities that are pre-arranged with car owners in the community (Table 2).

These findings led to a 6 scenario spatio-temporal model of access to care, that depicts the common modes of transport from the community to PHCs and through the facility referral network, including;

1. Walking to PHCs
2. Public transport to PHCs
3. Walking to PHCs and driving to SHFs (using ambulances)
4. Public transport to PHCs and driving to SHFs (using ambulances)
5. Walking to PHCs, driving to SHFs and driving to THFs (using ambulances)
6. Public transport to PHCs, driving to SHFs and driving to THFs (using ambulances).

## Results

### Seasonal variation in travel times to health facilities

**Fig. 5:**
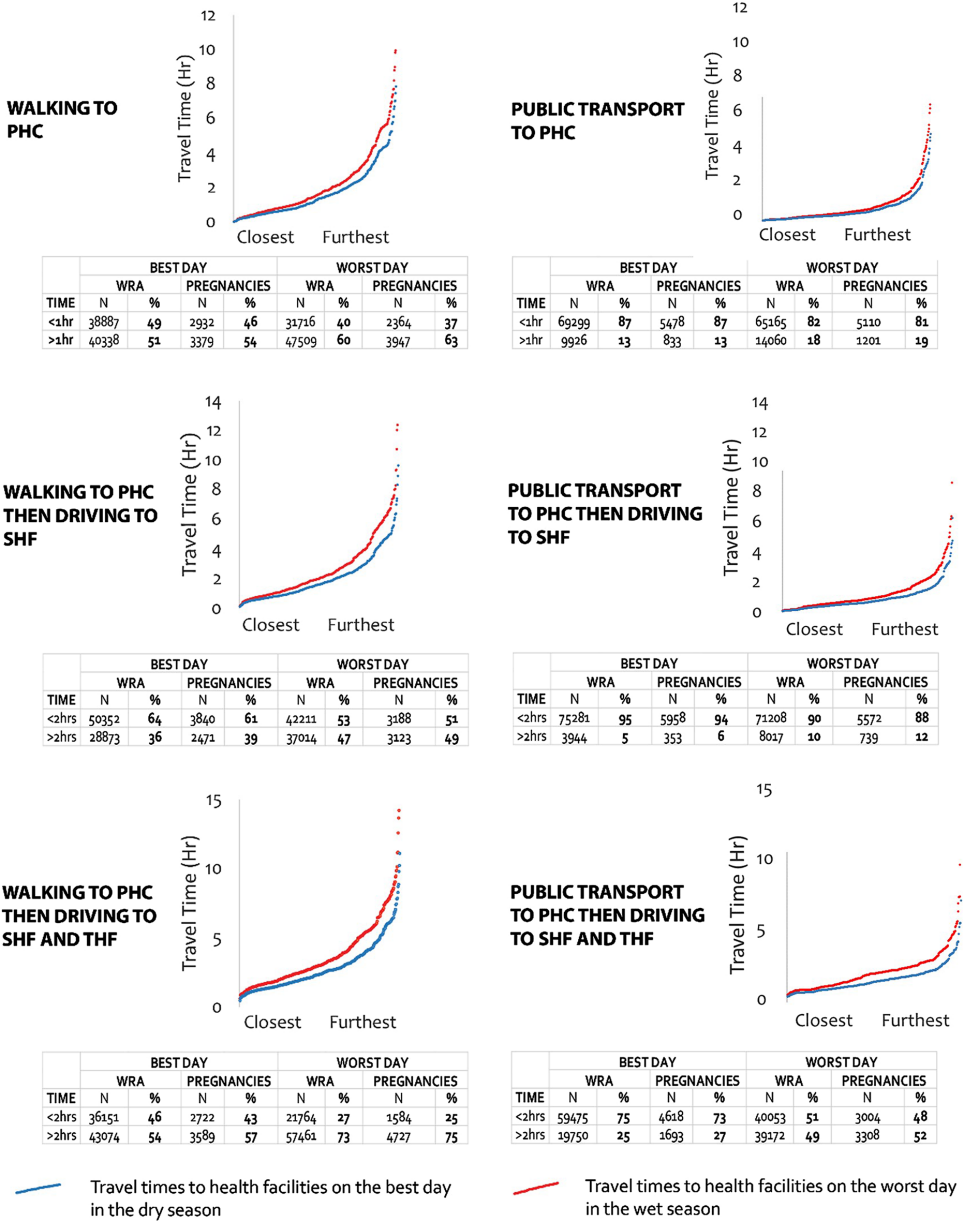
Travel times using different transport modes and percentage of women within facility’s catchments

Spatial access to health services generally decreases during the wet season for all modes of transport and to all level facilities due to the increase in travel times that are imposed by precipitation and flooding. At the peak of the dry season, 49% (*n* = 38,887) of women of reproductive age included in the census lived within a 1-h walking time to the nearest PHC (Fig. 5). Approximately 6311 of women of reproductive age, would have been pregnant at the time, with 46% (*n* = 2932) also living within 1-h walk from a PHC. The population of women of reproductive age within a 1-h walking time to PHC dropped by 9 to 40% (*n* = 31,716) while that of pregnant women dropped by 11 to 37% (*n* = 2364) at any time during the wet season. The furthest communities were up to 7.9 h walking time to PHC during the dry season. However, this increased to 9.9 h at the peak of the wet season.

A similar pattern of reduced access is observed for travel to other level facilities for women that began their journeys with walking to PHCs. At the peak of the dry season, 64% (*n* = 50,352) and 46% (*n* = 36,151) of women of reproductive age lived within 2-h travel time to the nearest SHF and THF, respectively, that offer life-saving maternal care. Approximately 61% of the women of reproductive age (*n* = 3840) and 43% (*n* = 2722) of pregnant women also lived within 2-h travel time to SHF and THF, respectively. Populations of women of reproductive age living within 2-h from SHF and THF dropped to 53% (*n* = 42,211) and 27% (*n* = 21,764), respectively, while that of pregnant women dropped to 51% (*n* = 3188) and 25% (*n* = 1584), respectively, during the rainy season because of weather induced increases in travel in travel time.

The model scenarios involving the use of public transportation markedly reduce the travel times to all level facilities. At the peak of the dry season, 87% (*n* = 69,299) of women of reproductive age lived within a 1-h travel time to the nearest PHC using public transport. Approximately 87% (*n* = 5478) of pregnant women also lived within 1-h travel to the nearest PHCs. The population of women of reproductive age within 1-h travel time by public transport to PHC dropped by 5 to 82% (*n* = 65,165) while that of pregnant women dropped by 6–80% (*n* = 5110) at any time during the wet season. The furthest communities were up to 4.9 h of travel time to PHC using public transport during the dry season. However, this increased to 6.6 h at the peak of the wet season.

At the peak of the dry season, 95% (*n* = 75,281) and 75% (*n* = 59,475) of women of reproductive age lived within 2-h travel time to the nearest SHF and THF respectively. Approximately 94% (*n* = 5958) of the women of reproductive age and 73% (*n* = 4618) of pregnant women also lived within a 2-h travel time to SHF and THF, respectively. These populations of women of reproductive age living within two hours from SHF and THF dropped to 90% (*n* = 71,208) and 51% (*n* = 40,053), respectively, while that of pregnant women dropped to 88% (*n* = 5572) and 48% (*n* = 3004), respectively, during the rainy season.

There is a near exponential increase in travel times between the communities that are closest to health facilities compared to the ones that are the furthest (Fig. 5), indicating that huge disparities exist in access to maternal health services. For the furthest communities, there is a 2.1-hour increase in travel time to PHC, 2.7 h to SHF and 3.1 to THF for journeys that commences with walking to PHCs in the wet season. This increase in time is equivalent to walking approximately 10 extra kilometers to the nearest primary facility. Similarly, travel time increases by 1.7, 2.5 and 2.8 h to PHC, SHF and THF respectively are also observed for the furthest communities when journeys commence with public transport.

**Fig. 6:**
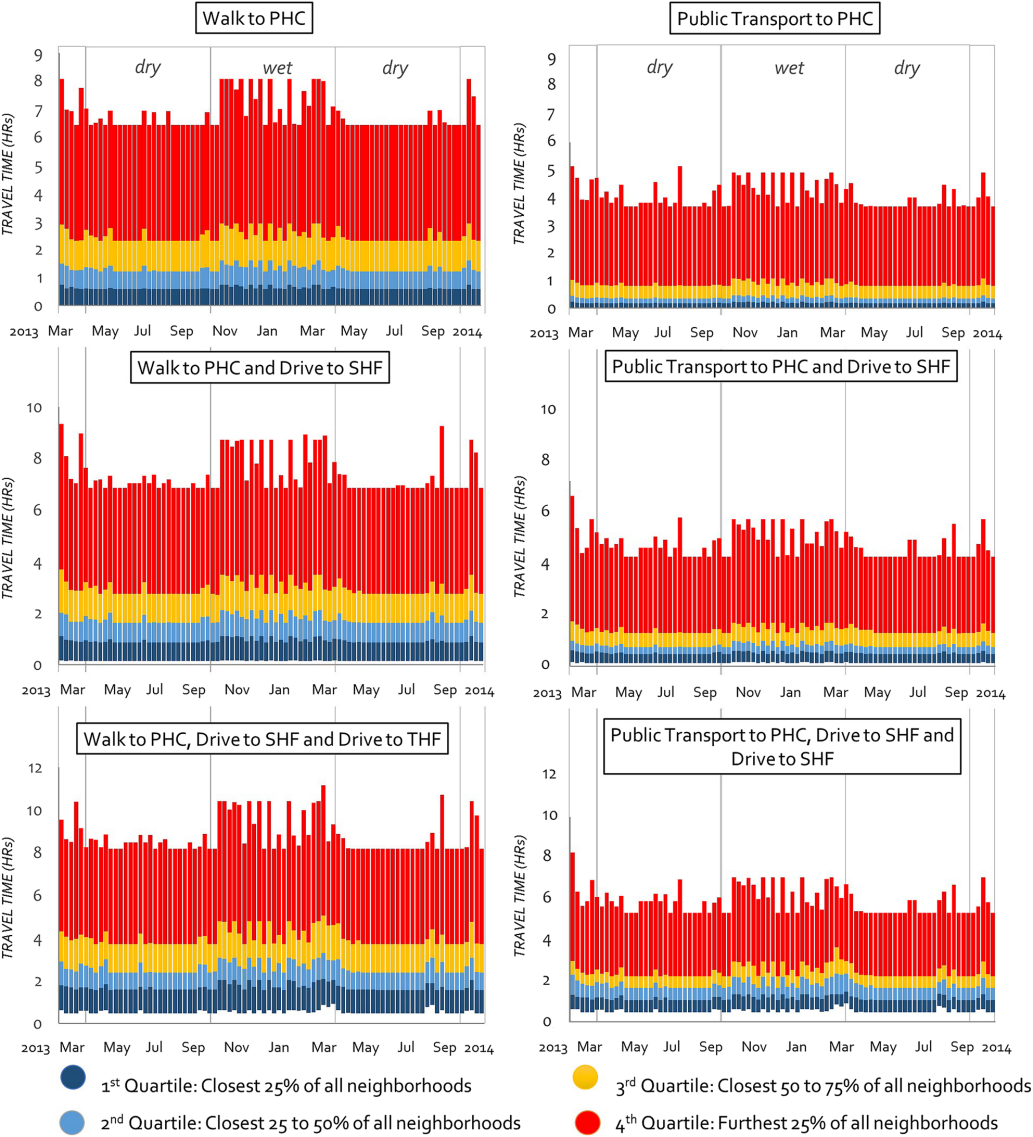
Seasonal variation in travel times for different modes for the 17-month timeline

The results suggest that the longer the travel time to a health facility the more likely populations are to be affected by precipitation or flooding (in and out of season)—hence the greater degree of fluctuation in travel times, particularly for the furthest communities in the fourth quartile of travel times (Fig. 6). While there are more stable travel times to health facilities during the dry season, there are minor fluctuations during this time resulting from the few instances of rainfall in the dry season. Figure 6 illustrates the extent to which the furthest communities are disproportionately isolated (and potentially disproportionately more vulnerable) as the upper quartile of communities (red colour) has a much larger range when compared to the 1st, 2nd and 3rd quartiles for all modes of transport and facilities.

**Fig. 7:**
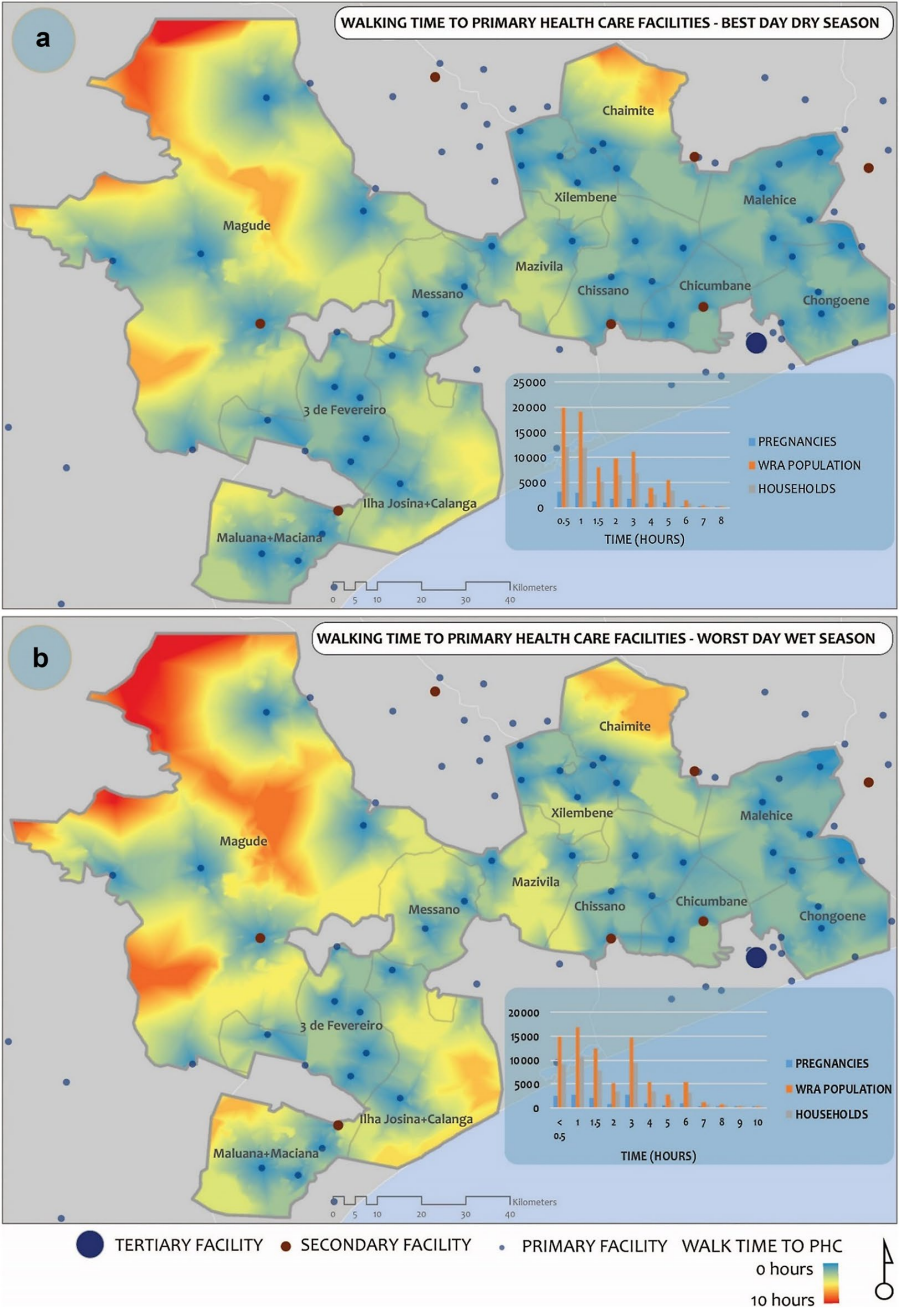
A comparison of walking times to the nearest PHC on the best day in the dry season (**a**) and worst day in the wet season (**b**)

In terms of the place specific impact of severe wet weather on access, it appears Magude district, Ilha Josina + Calanga and Chaimite regions are the areas most affected by the wet weather (Fig. 7), as they appear redder and yellower in B (wet weather) when compared to A (dry weather) for walking to PHCs. The reason for this is the low density of facilities as well as the poor road infrastructure in these regions. Populations in these regions will generally need to travel long distances on bad roads to access care. This is obviously aggravated by wet weather and flooding. The full set of maps indicating dry/wet weather differences in travel times have not been included but are available upon request. Some of spatio-temporal animations can be accessed at https://pre-empt.cfri.ca/monitoring/mapping-outcomes-mothers-mom.

**Fig. 8:**
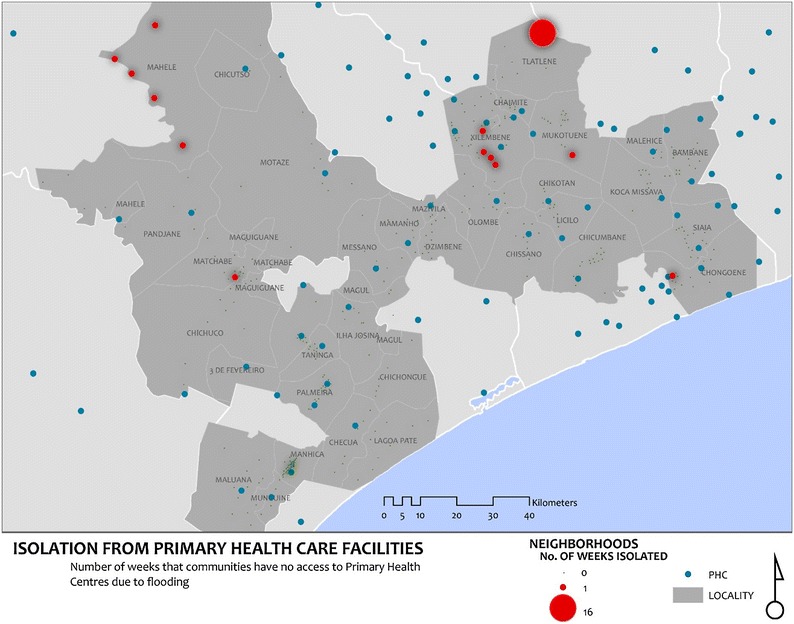
Communities isolated from care as a result of flooding

Thirteen of the 417 neighbourhoods were at some point completely isolated from health facilities (travel time > 99,999,999) during the study; 12 for one week and 1 for 16 weeks (Fig. 8). Most of these neighborhoods are unsurprisingly located in the regions of the study area shown in Fig. 7 to have been most severely affected by the wet weather.

## Discussion

This paper describes a new approach for measuring and visualizing spatio-temporal access to maternal health services. To our knowledge this is the first time that empirical records of precipitation have been incorporated into modelling spatio-temporal variation in access to care. Specifically, this work extends the current models of geographical access to maternal care by accounting for the multiple transport options that characterize women’s’ journeys, and how their transit changes depending on season.

The transport information provided in the CLIP facility assessment and baseline census describe what a typical woman’s journey from home to primary care facilities and through the rest of the health facility referral network looks like. While [34] demonstrated a model of spatial accessibility that accounts for mechanized and non-mechanized modes of transport, simulations of access were done for each mode in isolation. Other studies have projected travel times through the health facility referral chain in a hierarchical fashion similar to this study [27], but have overlooked the multiple transport options (walking, public transport, and use of ambulances) that characterize women’s journeys. Our study explored travel times that result from the use of mixed transport modes through the various tiers of the health care system and advances spatial modeling of access to maternal care in a direction more suited for the daily realities of women in low- and middle-income countries where vulnerability to poor pregnancy outcomes is highest.

While a previous study [14] examined the potential impact of seasonality on access to maternal health services, our use of empirical daily records of precipitation and floods has demonstrated that the seasonal variation in access cannot simply be imagined through a dichotomous, and static lens of wet and dry seasons, as access continually fluctuates. The elements do not only slow down travel to health facilities, but in some instances can isolate whole communities from accessing these services [26]. The use of real weather records enhanced our understanding of how access to maternal care may be hampered. Media sources confirm that the communities identified as having been isolated because of floods, were actually flooded, in some instance resulting in the community members being evacuated [44–46].

While these results indicate a sizeable reduction in the number of women who live within 1-h of basic care (PHC) and 2-h from life-saving care (SHF and THF) because of precipitation and floods, the transport mode used has a much greater impact on increasing travel times to health facilities. For example, pregnant women living within an hour of primary care facilities are shown to increase by 41% when women access public transport from their community, compared to when they walk to PHCs to seek care (Fig. 5). A similar pattern exists for SHFs with an increase of 33% for pregnant women who live within 2-h, resulting in 95% of all women living within 2-h of life-saving maternal care. This significant reduction in travel times because of access to public transport illustrates the potential impact of community-level transport related support for women needing to access maternal care, and further confirms the value of initiatives aimed at increasing access to transport as they will potentially greatly increase access to care both in the dry and wet seasons.

While the dominant view on geographical access to care assumes a need to measure distances or travel times from communities to health facilities, an emerging model of care in many low- and middle-income countries includes care by community health workers (CHWs) [47]. CHWs are “lay members of communities who work either for pay or as volunteers in association with the local health care system in both urban and rural environments and usually share ethnicity, language, socioeconomic status and life experiences with the community members they serve” [48]. These minimally trained workers extend the reach of basic health services in communities that are not well covered by health centres’ [49]. The impact of flooding on hindering community health workers from accessing to pregnant women in need of services have been reported by [50]. The blend of weather sensitive spatio-temporal model of access with the upcoming strategies for reaching the most isolated populations with health services through a mobile health force [51, 52] will potentially take to utility of spatio-temporal models of access beyond macro-planning of health services and make them operational on a daily basis at the community-level. The increased recognition of community level health surveillance, including pregnancy surveillance and mobile health technologies [52, 53] will set the context where these daily pictures of access could inform decisions by community health workers as they link communities to formal health services.

A limitation to our approach to modelling spatio-temporal access is that it does not account for wait times at facilities, as these data were not available. Wait times would provide a more accurate picture of how long it takes to navigate through the health care systems, accounting for both geographical and health services related delays. Including waiting times in the modelling process would be a step closer to modelling all three delays of triage, transport and treatment [54]. Further to that, in modelling the use of public transport, we did not account for time a woman may need to wait to get a vehicle once she gets to the main road. Waiting for transport is a known barrier to care seeking in the study area [25], thus making travel time estimate very conservative.

Another limitation for this study pertains to estimates for speed and travel time. While the ones used in this study have been adopted from previous studies on access to care, there is a lack of good evidence that these estimates are relevant for our study setting. There is also a lack of empirical data on the real extent to which precipitation reduces travel speed on different road types under study. Furthermore, safety concerns for women who may walk to seek emergency care at night, and the unavailability of public transport during evenings, have been previously noted as barriers for accessing to maternal care [15]. Future research is therefore needed on methods of generating empirical data for validating speed limits and how precipitation more precisely affects travel in different seasons and at different times of day.

The ideas and methods proposed in this paper can be translated to other health disciplines and settings where seasonal elements affect access to care. Similar problems of harsh weather impeding access to health care for geographically isolated regions in Aboriginal communities of Northern Canada [55]. Data show that women living in these communities are disproportionately more vulnerable and more likely to experience adverse maternal outcomes when compared with the rest of the Canadian population [4]. The proposed approach could be used to imagine new models of access that cater to the geography of, and temporal patterns in precipitation affecting women in these remote regions.

## Conclusions

Models for spatio-temporal access that account for the daily realities of women’s transport options in their communities are increasingly necessary. Understanding populations’ geographical access to maternal health services and how it varies by season will enable health services planners to better identify populations that are underserved by their spatial configuration, and best increase access to health care. Flooding and heavy rainfall continue to be apparent characteristics of climate change, that are known to impede access to maternal care. This study highlights how to combine daily records on precipitations and floods to enhance the understanding of the seasonal variation in spatial access to maternal care in a way that has an impact, not only on long-term planning of maternal health services but potentially on improving daily planning concerning access to care at the facility and extending the reach of care to the community. Initiatives that for transport support at the community level will complement the understanding of these spatio-temporal dynamics to accessing maternal care and will help women get to health facilities quicker.

## Abbreviations

CENACARTA: National Cartography and Remote Sensing Centre
CHW: community health worker
CLIP: community level intervention for pre-eclampsia
FEWSNET: Famine Early Warning Systems Network
GIS: geographical information systems
GPS: global positioning system
PHC: primary health centre
SHF: secondary health facility
THF: tertiary health facility
